# Association of an expanded inflammatory mediators response with clinical and laboratory data in the postoperative period of pulmonary resection: a prospective clinical study

**DOI:** 10.1590/0100-6991e-20213008

**Published:** 2021-11-05

**Authors:** LUCIARA IRENE DE NADAI DIAS, VALESKA DALANEZI PAULINO LEITE, JÚLIA MENDES BRANDÃO, ANDREA PELICIA ROSO, ELIANA CRISTINA MARTINS MIRANDA, EDSON ANTUNES, RICARDO KALAF MUSSI

**Affiliations:** 1 - Unicamp, Cirurgia Torácica - Campinas - SP - Brasil; 2 Unicamp, Farmacologia - Campinas - SP - Brasil

**Keywords:** Inflammation, Thoracic Surgery, Lung, Interleukins, Inflammation Mediators, Inflamação, Cirurgia Torácica, Pulmão, Interleucinas, Mediadores da Inflamação

## Abstract

**Introduction:**

patients undergoing pulmonary resection may experience local or remote complications in the postoperative period due to the inflammatory response, which increases the length of hospital stay and costs. This study objective was to establish an expanded interleukins profile, identifying the main actors in the postoperative inflammatory response, and to correlate them with clinical and laboratory data of patients submitted to pulmonary resection.

**Methods:**

this was a prospective, interventional, longitudinal study of 27 cases of pulmonary resection performed at HC-UNICAMP, in which we analyzed serum levels of IL 1 α, IL 1 β, IL 1 ra, IL 2, IL 13, IL 6, IL 8, IL 10, IL 12 (p40), IL 12 (p70), IL 17a, TNF α, TNF β, IFN γ, TGF β, MIP 1α, MIP 1β, MCP 1, MCP 3, VEGF, and clinical data before, during, and after surgery.

**Results:**

Individuals had a median age of 63 years, 16 (59%) being male and 11 (41%), female. The clinical factors that influenced inflammatory response were body mass index, smoking, and previous use of corticosteroids, while the influencing laboratory data were the numbers of leukocytes and platelets. **Discussion:** within this expanded interleukin profile in the inflammatory response of lung resections, our study showed that interleukins IL 6, IL 8, IL 10, IL 1 β, and TNF α should be considered for assessing humoral inflammation.

**Conclusion:**

this study can aid in the identification of clinical or pharmacological interventions that modulate the inflammatory response in the perioperative period of pulmonary resections, mitigating local and systemic complications.

## RESUMO

**Introdução:**

pacientes submetidos a ressecção pulmonar podem apresentar complicações locais ou remotas no pós-operatório decorrente da resposta inflamatória, que aumenta o tempo de internação e de custos hospitalares. O objetivo deste trabalho foi estabelecer perfil ampliado do comportamento das interleucinas, identificar as principais interleucinas que atuam na resposta inflamatória no período pós-operatório e associá-las com dados clínicos e laboratoriais dos pacientes submetidos a ressecção pulmonar.

**Métodos:**

Estudo prospectivo com 27 casos de ressecção pulmonar realizados no HC-UNICAMP. Foram analisados níveis séricos de IL-1 α, IL-1 β, IL-1 ra, IL-2, IL-13, IL-6, IL-8, IL-10, IL-12 (p40), IL-12 (p70), IL-17a, TNF- α, TNF- β, IFN-γ, TGF- β, MIP-1 α, MIP-1 β, MCP-1, MCP-3, VEGF e dados clínicos antes, durante e após a operação.

**Resultados:**

indivíduos apresentaram mediana de idade de 63 anos, sendo 16 (59%) do sexo masculino e 11 (41%) do sexo feminino. Os fatores clínicos que influenciam na resposta inflamatória são: índice de massa corporal, tabagismo e uso prévio de corticóide, enquanto que os dados laboratoriais se expressam nos números de leucócitos e plaquetas.

**Discussão:**

dentro deste ampliado perfil das interleucinas na resposta inflamatória das ressecções pulmonares, este estudo mostrou que devem ser valorizadas para avaliar inflamação humoral as interleucinas: IL-6, IL-8, IL-10, IL-1 β e TNF- α.

**Conclusão:**

este estudo pode colaborar na identificação de intervenções clínicas ou farmacológicas que modulem a resposta inflamatória no período peri-operatório das ressecções pulmonares, mitigando as complicações locais e sistêmicas.

## INTRODUCTION

Surgery is the standard treatment for early-stage neoplasms. In addition to malignant neoplasms, other lung diseases may require surgical treatment, such as those of benign neoplastic, inflammatory, and infectious, which can be treated by pulmonary resection^1^.

Surgical trauma is a well-known causal factor of local and systemic inflammatory response, which results in increased circulating levels of IL-6 and IL-8 and may play an important role in the development of postoperative complications^2^.

Acute lung injury (ALI), a direct expression of inflammation in this organ, can lead to acute hypoxemic respiratory failure or Acute Respiratory Distress Syndrome (ARDS) when intense. ALI and ARDS represent the spectrum of lung lesions, and the term ARDS is reserved for patients with more severe alterations in gas exchange^3^. The arbitrary cutoff line between ALI and ARDS may have clinical relevance, as patients with more severe lung injury often display simultaneous damage to other organs^4^.

Despite recent advances in diagnosis and treatment, Systemic Inflammatory Response Syndrome (SIRS) and sepsis are still associated with 25-60% of deaths in critically ill patients. After thoracic operations, the incidence of SIRS and postoperative sepsis increases considerably. Finding predictive markers of SIRS is a great challenge and some studies have been dedicated to this end^5^.

Therefore, the objective of this study was to establish an inflammatory response interleukin profile in the postoperative period and to correlate it with clinical and laboratory data from patients submitted to pulmonary resection.

## METHODS

We conducted a prospective, single-center study, which was approved by the Ethics in Research Committee (CEP) of the Faculty of Medical Sciences (FCM), State University of Campinas (UNICAMP) opinion n° 933/2009. The study followed the principle of equality and applied the Free and Informed Consent Term (TCLE) to all patients.

The procedures were performed between January 2012 to June 2014 at the Hospital das Clínicas (HC) of UNICAMP by the staff of the Thoracic Surgery Discipline, and the patients underwent pulmonary segmentectomy, lobectomy, bilobectomy, or pneumonectomy. Individuals could have been submitted or not to previous chemotherapy and/or radiotherapy, and if so, there was a minimum three-month period required before the procedure. In addition, they should have hemodynamic stability, without the use of vasoactive medications or metabolic and electrolyte alterations before and after the operation, without continuous or intermittent sedation after the operation, and without ventilatory support in the postoperative period. All procedures were performed in the morning. For the evaluation and comparison of the magnitude of the inflammatory response after pulmonary resection, patients were their own controls, with data from the preoperative period.

We collected demographic and clinical data directly from medical records, while the anesthesia team provided information on the intra- and postoperative analgesia administration mode, type of intubation, ventilatory mode, monopulmonary ventilation time, tidal volume, PEEP and the inspired oxygen fraction delivered to the patient, need for vasoactive drugs, blood transfusions and / or crystalloids infusion, ischemia time if present, operation time, and extubation site.

No patient displayed criteria for exclusion from the research, ie postoperative hemodynamic instability, renal failure, pulmonary embolism, aspiration pneumonia, or ventilatory disorder.

In the pre and postoperative period, we analyzed leukocytes, platelets, IL-1 α, IL-1 β, IL-1 ra, IL-2, IL-13, IL-6, IL-8, IL-10, IL-12 (p40), IL-12 (p70), IL-17a, TNF-α, TNF-β, IFN-γ, TGF-β, MIP-1 α, MIP-1 β, MCP-1, MCP-3, VEGF, arterial blood gas (PaO2), oxygenation index, peripheral saturation, respiratory rate (RR), heart rate (HR), and body temperature.

We also assessed the oxygenation index and PEEP. We obtained the peripheral saturation index by means of a pulse oximeter, and the respiratory (RR) and heart (HR) rates by a non-invasive monitor, all adapted to the patient to measure vital signs.

We collected samples for arterial blood gases preoperatively, at the exact time when bipulmonary ventilation was resumed, in the immediate postoperative period (IPO), and at four, eight, 24, and 48 hours postoperatively.

We harvested venous blood samples preoperatively, in the IPO, and at four, eight, 24, and 48 hours postoperatively. One portion was sent to leukocyte count and other was centrifuged and cooled at -80° C, separated from the plasma, and submitted to measurements of levels of IL-1 α, IL-1 β, IL-1 ra, IL-2, IL-13, IL-6, IL-8, IL-10, IL-12 (p40), IL-12 (p70), IL-17a, TNF α, TNF β, IFN-γ, TGF β, MIP-1 α, MIP-1 β, MCP-1, MCP-3, and VEGF, using kits for Multiplex analysis (micro-assays).

### Statistical analysis

We performed a descriptive statistical analysis, then applied the paired t test and analysis of variance associated with Bonferroni test, considering the p-value <0.05 as significant. The software used was the Statistical Package for Social Sciences (SPSS) version 24.0 (SPSS Statistics; IBM, Armonk, NY, USA).

We did not carry out a sample size calculation because this was a pilot therapeutic study (phase II), whose purpose was to try to establish the time and cause of the inflammatory response, and therefore to predict which patients would have the worst evolution and propose the necessary measures to minimize the outcome. For this, we decided to do a prospective, single-center study, comprising patients undergoing complex, high-risk procedures. It was then necessary to estimate the period for the study and not the number of cases. Following the principle of equality, with the results in hand, one can establish new conducts that directly promote new interventions and randomized studies at the same time. 

Importantly, the p value was not corrected for multiple analyses, which increases the probability of spurious associations.

## RESULTS

We included 27 patients, with a median age of 63 years (29 80), 16 (59%) being male, 89% white. The distribution of the baseline diagnosis was 29.5% adenocarcinoma and 29.5% carcinoma, and 15% of cases presented with metastases (Table 1).

**Table 1:** Characteristics of the 27 cases.

Variables	n=27	%
Male	16	59
White skin color	24	89
Age, years (average, range)	63 (29-80)	
BMI Calculated, kg/m^2^ average	24,8 (± 13)	
Smoker / ex-smoker	6/8	22,0/29,5
Previous use of corticosteroids	5	18,5
Previous use of bronchodilators	10	37,0
Basic diagnosis		
Adenocarcinoma	8	29,5
Carcinoma	8	29,5
Aspergilloma	3	11,0
Liposarcoma	2	7,5
Lung tumor to be clarified	2	7,5
Others*	4	15,0
Main associated diseases**		
Hypertension	7	26,0
OPDC	6	22,0
Diabetes mellitus	4	15,0

BMI Calculated: body mass index. *1 case of myxoid sarcoma; 1 bronchiectasis; 1 lobectomy for the after math of a gunshot wound and suture; 1 Cystic adenomatoid. **There are cases with more than one disease; 2 Deep vein thrombosis; 1 Benign prostatic hyperplasia; 1 Epilepsy; 1 hypothyroidism; 6 alcoholism.

The main data of the surgical procedure are presented in Table 2. A patient was extubated in the ICU, and 26 (96%), in the operating room.

**Table 2:** Surgical procedure data.

Variables	n=27	%
Types of surgeries		
lobectomy	18	67
Segmentectomy	04	15
Bilobectomy	02	7,5
Pneumonetectomy	01	3,5
Wedge Resection / Lipoma Removal	02	7
Surgery site		
Upper lobe	12	44,5
Lower lobe	12	44,5
Both	03	10,0
Right side	17	63,0
Selective intubation	26	96,5
Types of ventilation		
Controlled pressure (CP)	17	63
Controlled volume (CV)	10	37
*Intraoperative volume, ml	400 (200-1500)	
*Single lung ventilation time, h	3 (1-4,5)	
*Duration of surgery, h (average, range)	4,5 (3-6,5)	

Regarding the association of variables/pre-determining factors for the inflammatory response, body mass index (BMI), smoking, associated diseases, and previous use of corticosteroids showed statistical significance (Table 3).

**Table 3:** Correlation of Interleukins and Clinical Data.

Interleukin	Clinical data		
IL-10	BMI Calculated	p<0,05	R^2^ de 40 a 49%
IL-1 β	BMI Calculated	p<0,05	R^2^ de 40 a 49%
IL-6	Smoking	p<0,05	R^2^ de 46 a 50%
IL-8	Previous use of corticosteroids	p=0,02	R^2^ -45%
TGF- β	Previous use of corticosteroidsm	p=0,006	R^2^ de 51%
MCP-3	BMI Calculated	p<0,05	R^2^ de 40 a 53%
IL-12 (p40)	BMI Calculated	p=0,02	R^2^ de 43%
IL-1 ra	Previous use of corticosteroids	p<0,05	R^2^ de -41 a -53%
MCP-1	BMI Calculated	p=0,03	R^2^ de -41%

We observed that the higher the BMI, the higher the found concentration of IL-10, IL-1 β, MCP-3, and IL-12 (p40), and the lower the concentration of MCP 1. The older the patient, the higher the concentration of IL-8, TNF α and IL-1 ra. In smokers or ex-smokers, the concentration of IL-6 was higher.

Interleukins IL-1 α, IL-13, MIP-1 β, IFN-γ, and VEGF showed no association with the clinical data.

We present the graphs (1-5) of the pre- and postoperative behavior of the main interleukins’ profiles below:

**Graphic 1:**
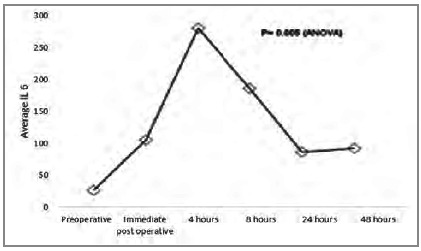
Average IL-6 pre and post surgery.

**Graphic 2 ch2:**
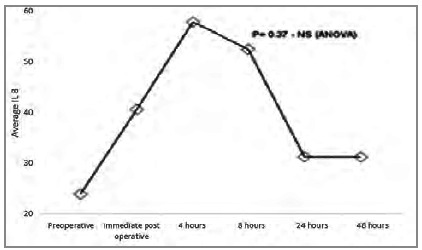
Average IL-8 pre and post surgery.

**Graphic 3 ch3:**
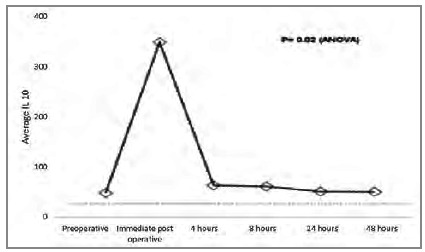
Average IL-10 pre and post surgery.

**Graphic 4 ch4:**
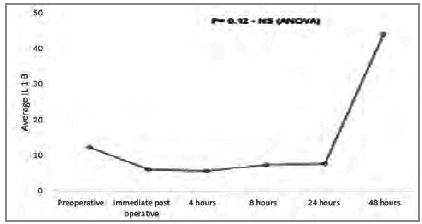
Average IL-1β pre and post surgery.

**Graphic 5:**
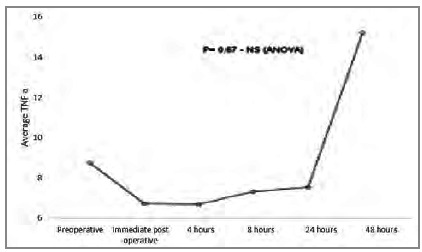
Average TNF-α pre and post surgery.

The leukocyte count had a significant decline after four hours of operation, as shown in Graphic 6. The mean number of platelets showed a decrease soon after the procedure (Graph 7).

**Graphic 6:**
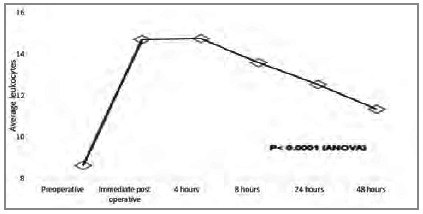
Average pre and post surgery leukocyte counts.

**Graphic 7:**
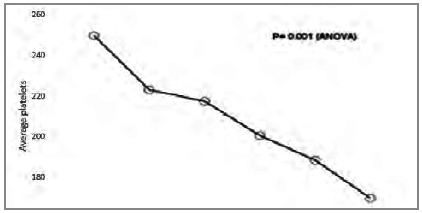
Average pre and post surgery platelets.

## DISCUSSION

### Inflammation

Many authors have analyzed predictive markers of mortality and morbidity in patients undergoing pulmonary operations, but reviews of inflammation and biomarkers are generally not included^6^ ^-^ ^8^. The proposed biological association between mediators and clinical and laboratory variables in this study resulted in interesting data, especially since we carried out a complete analysis of 20 analytes/markers, which we did not find in the literature.

According to Matesanz et al.^9^, the determination of cytokine levels allows the detection of an early inflammatory response. Therefore, it is important to know the base level of inflammatory response and the interrelationship between pro and anti-inflammatory interleukins so that the body’s response to different aggressions is modulated and the defense system is not harmful to its own body. However, high plasma levels of cytokines after operations are related to complications and postoperative mortality, and better knowledge may allow strategies to mitigate the complications in the postoperative period. According to Gala^10^, the systemic proinflammatory state during and after pulmonary surgery is responsible for postoperative systemic complications. The authors claim the same is true for local inflammation. Thus, pulmonary inflammation has been associated with postoperative pulmonary complications. These authors consider TNF-α as the main marker for pulmonary inflammation, which we also observed in our study in the uncomplicated postoperative period.

The relation between pro and anti-inflammatory interleukins can be assessed using the proportion IL-6 / IL-10 or TNF-α / IL-10, which has been associated with prognosis^11^. This relationship can be explained by the fact that IL-10 is the main anti-inflammatory interleukin and regulates the production of other interleukins. We observed that the increase in IL-10 peaks is in the immediate postoperative period, with significant reduction four hours after surgery, remaining low for up to 48 hours. TNF-α remains low (probably by the action of IL-10) in the first postoperative hours, rising after 24 hours, reaching the peak 48 hours after surgery. The behavior of IL-6 also seems to be influenced by IL-10, as it starts to rise in the immediate postoperative period, peaks four hours after the operation (when the drop in IL-10 starts) and continues to decline for up to 24 hours after the procedure.

Leite et al.^12^ showed that after single-lung ventilation during surgery, re-expansion after bronchial occlusion for one or three hours promotes inflammatory responses in the right lung, characterized by edema formation, neutrophil recruitment, and increased myeloperoxidase (MPO) activity. These changes are accompanied by high levels of IL 6, IL-1 β and / or TNF-α in the bronchoalveolar lavage. In addition, local lung injury was accompanied by systemic inflammation, as detected by increased serum levels of IL-6 and IL-10. 

In our study, no patient had SIRS, different than the report by Takenaka et al.^13^, who found 16 SIRS occurrences among 45 patients. This may be explained by the difference in surgical methods, operating time, and amount of blood lost, which are indicators of postoperative complications. The authors found no statistical difference between the group with SIRS and the group without it as to the plasma levels of IL-6, suggesting that IL-6 may be more qualitative than quantitative in terms of inflammatory response.

The Multiplex we used proved to be an excellent analysis method, as it requires a smaller plasma sample and has greater sensitivity when compared to ELISA. As we only included patients without postoperative complications, the magnitude of the serum interleukin levels was low, and the sensitivity of the ELISA method was not sufficient to detect the low values. However, this has been possible by the Multiplex technique, which uses minimum plasma aliquots for the analysis of a broad humoral profile of 20 inflammatory markers. Another relevant point of this work was that it was able to identify variations of other interleukins in addition to those traditionally known (IL-1 β, IL-6, IL-8, IL-10) in the inflammatory response to resection, which may be useful in new research and therapeutic strategies, among these TGF-β, MCP-3, IL-12 (p40), IL-1 ra, and MCP-1. Other authors have successfully used the ELISA method, but with a lower number of interleukins evaluated, and the patients had complications, with a high rate of inflammation^14^ ^-^ ^16^.

Among the main findings of this study, we highlight the decrease in leukocytes and platelets, the relationship of serum levels of interleukins with BMI, related diseases (diabetes, hypertension, and COPD), smoking, and prior use of corticosteroids.

### Platelets and leukocytes

Luo and colleagues^17^ reported that the systemic inflammatory responses can seriously injure the lungs, stimulating efforts to explore how to mitigate the damage. These authors evaluated whether platelets could help attenuate lung injury in mice resulting from systemic inflammatory responses induced by cardiopulmonary bypass (CPB) and concluded that, under certain conditions, platelets can protect the lung from injury induced by systemic inflammatory responses. We also observed the decrease in these markers, with platelets falling from the immediate postoperative period on and leukocytes showing an initial rise and then a fall. The metanalysis of 12 studies by Peng et al.^18^ demonstrated that platelets and leukocytes are efficient biomarkers of inflammation.

### Body Mass Index (BMI)

Our results showed that the higher the patients’ BMI, the higher the levels of IL-10, IL-1 β, MCP-3, and IL-12 (p40), and the lower the concentration of MCP-1. This finding corroborates the data of Speretta et al.^19^ that indicated that adipocyte hypertrophy increases the production of proinflammatory adipokines, however contradicting a finding of the same study, in which there was a reduction in the production of anti-inflammatory adipokines, such as IL-10.

### Smoking

Bastin et al.^14^ suggested that the increase in plasma IL-6 is a known marker for systemic inflammation. Matesanz et al.^9^ reported that non-cardiac thoracic surgery triggers the inflammatory cascade, which depends on the disease, on the surgical approach, and on the use of mechanical ventilation during surgery, especially in patients with lung lesions from tobacco, but they did not associate it with any specific interleukin. Elisia et al.^20^ showed that plasma samples of 30 heavy smokers (16 men and 14 women) had significantly higher levels of IL-6 than 36 non-smoking individuals.

Our study showed that the concentration of IL 6 was higher in smokers or former smokers, which indicates that smoking influences the postoperative elevation of pro-inflammatory cytokines. This finding agrees with the ones of Mosson et al.^21^ (2018), who studied the concentrations of somatostatin and IL 6 during acute pancreatitis and concluded that they were significantly higher in smokers. Another study proved that the inflammatory marker IL-6 and VEGF levels were found to be high and the anti-inflammatory marker IL 10, low, in smokers^22^.

### Previous use of Corticosteroids

In this study, when patients previously used corticosteroids, concentrations of TGF-β were higher and the ones of IL-8 and IL-1 ra were lower, results that corroborate the ones from Ren et al.^2^, who concluded that IL-8, among other markers, can be used as a serum marker of glucocorticoid efficacy in bronchial asthma.

Choi et al.^24^ carried out a retrospective study with 58 patients and concluded that early initiation of corticosteroids improved lung damage in patients with ALI and was also beneficial for weaning from the mechanical ventilator after lung resection for lung cancer.

We found no studies like this in the literature, relating a broad spectrum of interleukins with postoperative clinical and laboratory data of patients submitted to pulmonary resection. Interleukins IL-1 α, IL-13, MIP-1 β, IFN-γ, and VEGF did not show any statistically significant association with clinical data in the present study. This data diverges from Ugur et al.^22^, who observed a serum increase in VEGF in smokers. Relationship with interleukins IL-1 α, IL-13, MIP-1 β, IFN- γ were not found in the literature.

Kaufmann et al.^25^ state that complications after pulmonary operations are frequent, which increases the length of stay and hospital costs. Several risk factors have been identified, but it is still difficult to predict which ones are associated with inflammation in post-pulmonary resection complications. With the evaluation in this study, it was possible to observe that obese patients and smokers are more likely to develop postoperative inflammation, even without surgical complications. Previous use of corticosteroids has been shown to be efficient in reducing postoperative inflammation. More studies are needed to confirm these results.

 The number of participants in this study was limited, thus the authors encourage conducting studies with higher numbers of individuals to establish profiles of interleukins in the inflammatory response following lung resections. This will allow for better understanding and, consequently, selective therapeutic interventions, which will favor shorter hospital stays and lower costs.

## CONCLUSION

The inflammatory response is multifaceted and not clearly elucidated. However, we conclude that the markers that should be appreciated are IL-6, IL-8, IL-10, IL-1 β, and TNF-α, as the other interleukins, when associated with clinical data, had no clinical relevance. We conclude that BMI, smoking, and previous use of corticosteroids are pre-determining factors of inflammatory response.
